# Development and validation of cost-effective SYBR Green-based RT-qPCR and its evaluation in a sample pooling strategy for detecting SARS-CoV-2 infection in the Indonesian setting

**DOI:** 10.1038/s41598-024-52250-w

**Published:** 2024-01-20

**Authors:** Ratika Rahmasari, Muhareva Raekiansyah, Siti Hana Aliyah, Priska Yodi, Fathan Baihaqy, Muhamad Irhamsyah, Kartika Citra Dewi Permata Sari, Herman Suryadi, Meng Ling Moi, Rani Sauriasari

**Affiliations:** 1 Microbiology and Biotechnology Laboratory, Faculty of Pharmacy, Universitas Indonesia, Depok, West Java Indonesia; 2 Helix Laboratory & Clinic, Depok, West Java, Indonesia; 3 Department of Microbiology, School of Life Sciences & Technology, Institut Teknologi Bandung, Bandung, West Java Indonesia; 4 Clinical Pharmacy and Social Pharmacy Laboratory, Faculty of Pharmacy, Universitas Indonesia, Depok, West Java Indonesia; 5 School of International Health, Graduate School of Medicine, The University of Tokyo, Tokyo, Japan

**Keywords:** Biological techniques, Biotechnology, Molecular biology

## Abstract

A low-cost SYBR Green-based RT-qPCR method to detect SARS-CoV-2 were developed and validated. Primers targeting a conserved and vital region of the N genes of SARS-CoV-2 were designed. *In-silico* study was performed to analyse the compatibility of the selected primer pair with Indonesian SARS-CoV-2 genome sequences available from the GISAID database. We determined the linearity of our new assay using serial dilution of SARS-CoV-2 RNA from clinical samples with known virus concentration. The assay was then evaluated using clinically relevant samples in comparison to a commercial TaqMan-based test kit. Finally, we applied the assay in sample pooling strategies for SARS-CoV-2 detection. The SYBR Green-based RT-qPCR method was successfully developed with sufficient sensitivity. There is a very low prevalence of genome variation in the selected N primer binding regions, indicating their high conservation. The validation of the assay using clinical samples demonstrated similar performance to the TaqMan method suggesting the SYBR methods is reliable. The pooling strategy by combining 5 RNA samples for SARS-CoV-2 detection using the SYBR RT-qPCR methods is feasible and provides a high diagnostic yield. However, when dealing with samples having a very low viral load, it may increase the risk of missing positive cases.

## Introduction

The rapid transmission of severe acute respiratory syndrome coronavirus-2 (SARS-CoV-2), which caused the coronavirus disease 2019 (COVID-19) pandemic, had created a serious public health problem worldwide. The pandemic poses unprecedented challenges for health laboratories in conducting virologic testing with reliable results, as measures of diagnosis as well as in relation to public health. To meet this challenge, accurate and rapid diagnostic, and surveillance strategies for widespread use in different laboratory settings were needed^1,2^.

SARS-CoV-2 is an enveloped and non-segmented positive-stranded RNA virus belonging to the β-subgroup of the Coronaviridae family, which commonly infects humans and mammals^3^. Previously, this family of viruses has caused two epidemics: severe acute respiratory syndrome coronavirus (SARS) and Middle East respiratory syndrome coronavirus infection^4,5^.The SARS-CoV-2 genome is around 29.9 kb in length. It comprises 14 open reading frames (ORFs), encoding 16 non-structural proteins that constitute the replicase complex, nine accessory proteins (ORF), and four major structural proteins: the spike (S), envelope (E), membrane (M), and nucleocapsid (N)^6^. Among these proteins, the N protein is highly conserved in the CoV’s genus and is one of the most abundant structural proteins in virus-infected cells^7^.

Early diagnosis and isolation of suspected individuals plays a pivotal role in the containment of COVID-19. In addition, extensive screening testing must be implemented, particularly in affected areas. To meet these needs, increasing the capacity for testing and its availability is crucial^8^. A wide variety of diagnostic methods for SARS-CoV-2 infection have been developed, most of which rely on reverse transcription real time polymerase chain reaction (RT-qPCR) to detect the viral genomic RNA of the virus. Due to its high specificity and sensitivity, being able to detect minute amounts of nucleic acids, RT-qPCR is regarded as the gold standard for analysis and quantification of pathogenic RNA viruses in clinical samples, including SARS-CoV-2^9^. According to the World Health Organization (WHO), RT-qPCR methods using TaqMan probes are the current standard diagnostic assay for SARS-CoV-2 detection in samples mainly taken by smear (swab) from the nasal and oropharyngeal of suspected individuals. Since all the protocols recommended by WHO are based on the use of fluorogenic probes, these techniques are expensive and can be difficult to perform in low- and middle-income countries with a limited supply of reagents. The protocols also use different probes to target more than one gene of the virus, which involves additional cost. Therefore, low-cost diagnostic tests need to be developed as an alternative method for SARS-CoV-2 detection.

Several studies have attempted to develop RT-qPCR protocols using the double-stranded DNA binding dye SYBR Green. This method offers several advantages in its implementation over TaqMan chemistry, as it is cheaper and does not require the synthesis of specific probes. In contrast to TaqMan methods, in which fluorescence emitted from reporter dyes are conjugated to the specific oligonucleotide sequences of the probes, SYBR Green intercalates into any double-stranded DNA and emits a positive signal. Consequently, the main disadvantage of the SYBR Green method is that the dye can bind to any double-stranded DNA, including unspecific PCR products and primer dimers, which can lead to false positive signals. However, the accuracy of the SYBR Green-based method can be verified through melting curve analysis at the end of PCR. Through this analysis it can be ensured that only specific targets are amplified^10^.

Another approach to rapidly increasing the SARS-CoV-2 testing capacity of laboratories is the pooled screening strategy. This approach might also limit the use of reagents, increasing overall testing efficiency and thus contributing to early patient assistance and control of the pandemic. In such a pooling method, multiple specimens are combined in a single test. If negative pooling is detected, all samples in the pool are assumed to be below the test’s detection limit. In contrast, if positive pooling is detected, each specimen is required to undergo further individual evaluation^11^.The pooled screening strategy could be implemented most effectively in low prevalence settings with a 5–6% positivity rate of infection^12–14^. Numerous studies have demonstrated that the pooling testing method results in high agreement on positive specimen detection and a comparable cycle threshold (Ct) value between pooled and individual specimens, although an expected slight loss of sensitivity has been shown, particularly when individual specimens have a low viral load^15–17^.

The study aimed (i) to develop and validate an alternative RT-qPCR protocol for detecting SARS-CoV-2 from clinical samples by employing SYBR Green dye instead of fluorogenic TaqMan probes; and (ii) to evaluate the use of the SYBR Green-based RT-qPCR protocol in the sample pooling strategy with regard to the sensitivity of SARS-CoV-2 RT-qPCR tests. We showed that our developed SYBR Green-based RT-qPCR assay that targets the N gene is a reliable method for detecting SARS-CoV-2 in clinical samples, since the results were comparable to commercial TaqMan-based RT-qPCR. Furthermore, the pooling strategy demonstrated that combining up to 5 samples for SARS-CoV-2 detection using SYBR Green-based RT-qPCR analysis offers a high diagnostic yield. However, for samples having a very low viral loads, pooling may increase the risk of missing positive cases. Nonetheless, these results indicated that the pooling strategy is a reliable approach for efficient detection of the SARS-CoV-2 virus and could be implemented to increase the testing capacity for diagnosis and epidemiological surveillance purposes.

## Material and methods

### Primer design and in-silico primer analysis

**Figure 1:**
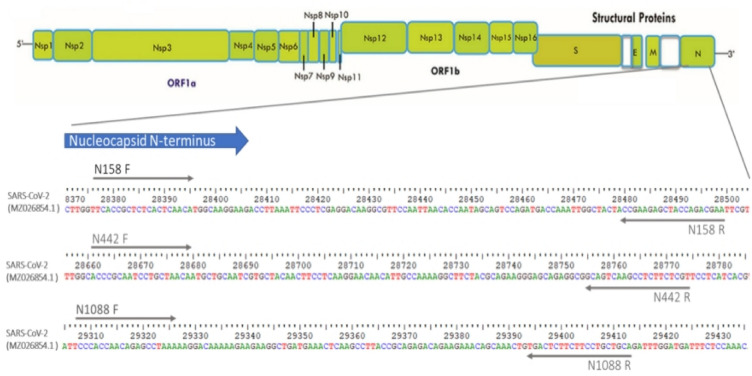
Location of the N-gene specific primers designed for the study in the SARS-CoV-2 genome.

The primers were designed based on available full sequence of SARS-CoV-2 virus from an Indonesian patient. The sequence was retrieved from the NCBI Reference Sequence Database (https://www.ncbi.nlm.nih.gov/nuccore/MZ026854.1). To design specific primers to target N genes, we utilized NCBI-Primer BLAST (http://www.ncbi.nlm.nih.gov/tolls/primer-blast). The forward and reverse primer sets were synthesized and delivered by Macrogene (Republic of Korea). The positions of each primer are shown in Fig. 1 and their sequences are listed in Table 1.

**Table 1:** Sequence of primers designed for SYBR Green-based RT-qPCR method.

Primer name	Sequences (5'-3')	Primer length (bp)	Product length (bp)	Annealing temp. (°C)	Accession number (bp)
N158 F	TCA CCG CTC TCA CTC AAC AT	20	123	59.03	_MZ026854.1_
N158 R	TTC GTC TGG TAG CTC TTC GG	20		59.19	
N442 F	ACC CGC AAT CCT GCT AAC AA	20	114	59.96	MZ026854.1
N442 R	ACG AGA AGA GGC TTG ACT GC	20		60.04	
N1088F	TCC CAC CAA CAG AGC CTA AA	20	107	58.56	MZ026854.1
N1088F	TGC AGC AGG AAG AAG AGT CA	20		58.95	
GADPH F	CAA TGA CCC CTT CAT TGA CC	20	155	61.68	_NM_002046.7_
GADPH R	TTG ATT TTG GAG GGA TCT CG	20		60.44	

For *in-silico* primer analysis, the selected primer sets were aligned with or compared to viral genome sequences of the SARS-CoV-2 virus which infected Indonesian individuals, using BioEdit Sequence Alignment Editor. Sequence variants or mismatches occurring in the primer binding regions and their frequency were calculated and recorded. For this analysis, the genome sequences were downloaded from the Global Initiative on Sharing All Influenza Data (GISAID) database^18^, which were obtained upon free registration (http://www.gisaid.org/). A total of 998 genome sequences, including Delta (n = 250), Omicron and it’s subvariants (BA.1, BA.2, BA.3, BA.4.1, BA.5, BF.5 and XBB.1; n = 748), were used for the *in-silico* analysis.

### Samples and ethical clearance

Residual, de-identified nasal and oropharyngeal VTM samples collected during routine diagnostic procedures were used in this study. The samples were randomly selected from our existing laboratory's sample collection, including both a positive sample group and a negative sample group. The study protocol was approved by the Research Ethics Committee of the Faculty of Medicine, University of Indonesia, with approval number 22–03-0327.

### Total nucleic acid extraction

A total of 300 uL of viral transport medium (VTM) samples from nasal and oropharyngeal swabs were extracted for total nucleic acid content. The extraction of RNA was performed on the TANBead Smart LabAssist-32 extraction system (Taiwan Advanced Nanotech Inc., Taiwan) according to the manufacturer’s protocol. The final elution volume was 100 uL for each sample. The nucleic acid was stored at −70 °C until used.

### SYBR Green-based and TaqMan-based RT-qPCR for SARS-CoV-2 detection

Quantitative reverse transcription PCR (RT-qPCR) was performed using the SensiFAST™ SYBR® No-ROX One-Step Kit (Meridian Bioscience®, USA) and commercial TaqMan-based 2019-nCoV Nucleic Acid Diagnostic Kit (Sansure Biotech, China). This kit detects the ORF1ab and N genes of the SARS-CoV-2 virus and the human RNase P gene as an internal control. The results were considered positive when the Ct values of both the ORF1ab and N genes were ≤ 40; otherwise, when these were > 40, the results were considered negative. For the SYBR Green-based RT-qPCR method, master mix SYBR Green and a specific primer designed for N genes were used. For this method, each 20 uL reaction contained 10 uL of master mix, 0.6 uL of primer (0.25 uM final concentration each), 3.8 uL of nuclease-free water and 5 uL RNA, with the following conditions: 45 °C for 10 min for reverse transcription, inactivation of reverse transcriptase at 95 °C for 2 min, pre-denaturation at 95 °C for 5 s, followed by 40–45 cycles of denaturation at 95 °C for 5 s, annealing and extension at 60 °C for 20 s. The TaqMan probe-based method was performed according to the manufacturer’s instructions. Each 20 uL reaction contained 9 uL of master mix (enzyme mix + 5 × buffer real-time PCR), 1 uL of primer, 5 uL of nuclease-free water, and 5 uL of RNA. The thermal cycling involved 50 °C for 20 min for reverse transcription, inactivation of reverse transcriptase at 95 °C for 3 min, and then 40 cycles at 94 °C for 10 s and 55 °C for 40 s. Non-template control was included in each RT-qPCR run as a negative control. The PCR runs were analyzed with MA-6000 software, version 1.1 (Molarray, China). Auto threshold was set in all assays to determine the threshold cycle (Ct).

### Melting curve analysis and gel electrophoresis

To verify the specific amplification of the SYBR Green-based RT-qPCR product, melting curve analysis was performed after each run. To this end, the fluorescence signal of each amplified product was recorded continuously as the temperature was increased from 65 to 95 °C, with acquisition of fluorescence data every 0.3 °C. In addition to the melting curve analysis, RT-qPCR product size was also confirmed by separation on agarose gel. For this purpose, amplified products were submitted for electrophoresis in a 2% agarose gel containing 0.01% v/v gel red (Biotium, USA) at 100 v for 40 min. The gel was then analysed in a UV transilluminator. The expected amplified fragments of the targeted N genes were between 132 and 159 bp.

### Analytical sensitivity of SYBR green-based assay

The analytical sensitivity of the assay was determined using seven serial tenfold dilutions of a SARS-CoV-2 positive RNA sample with a low cycle threshold (Ct). The sample was collected on November 8, 2022 from a 39-year-old female patient with Ct values of 15.61 for the ORF1ab gene and 12.30 for the N gene (tested by a Taqman-based commercial kit). The virus was identified as belonging to the Omicron lineage B.1.1.529 + BA variant (Accession ID: EPI_ISL_14754913; https://www.epicov.org). The RNA concentration of the original sample was measured by the methods described in our previous study^19^. All seven diluted samples were tested in triplicate by the SYBR Green-based RT-qPCR protocol. To generate the calibration curve, the resulting Ct values were plotted against the RNA concentration (RNA copy number) of the diluted standard. Efficiency (E) was calculated as E = 100 × (10 ^−1/s^–1), where s is the slope of the calibration curve. We also compared the SYBR Green-based and the TaqMan-based commercial RT-qPCR assay using 30 samples which had previously tested positive and negative.

### Pooling study

**Figure 2:**
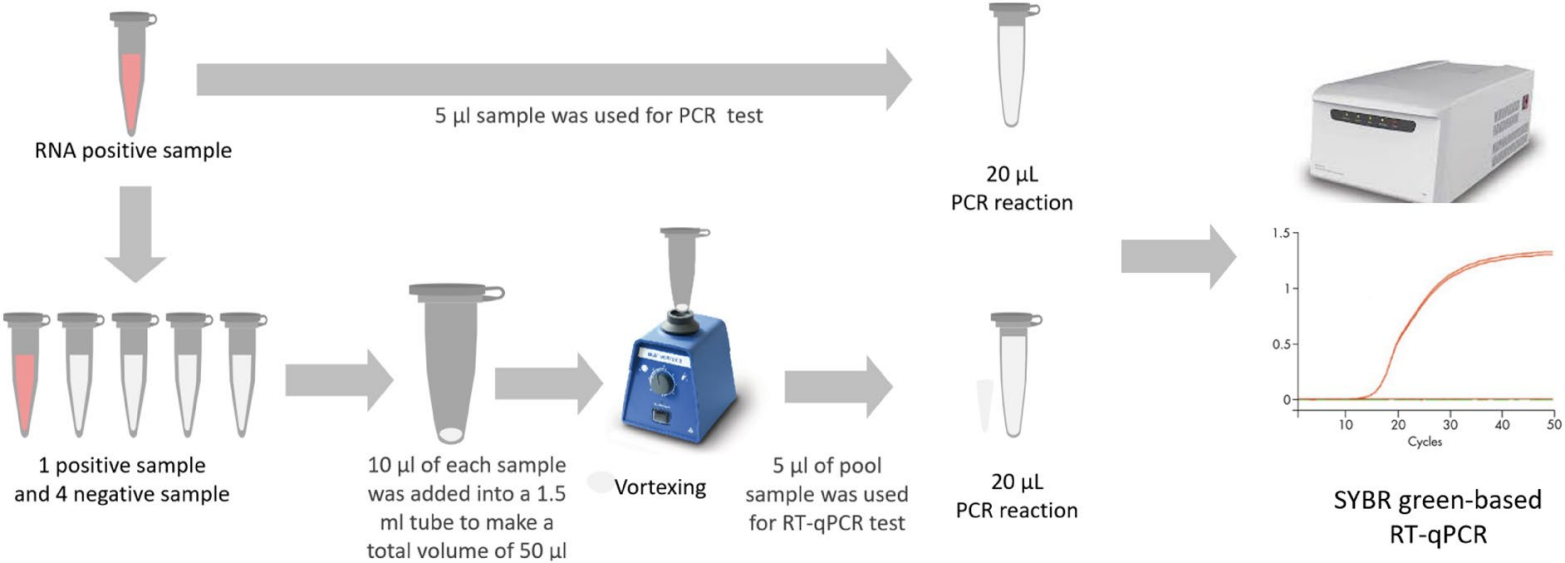
Schematic illustration of sample pooling technique of SARS-CoV-2 detection.

For the pooling study, 70 previously determined positive RNA samples were used, including 36 samples that had tested as weak positives with a Ct value greater than 30 (Ct ≥ 30). The 70 pools were prepared by mixing one positive sample with four confirmed negative samples, requiring a total of 350 negative samples to prepare these 70 pools. In each pool, equal volumes of 10 µl from one positive sample and four negative samples were combined in a 1.5 mL tube, resulting in a total volume of 50 µL. Subsequently, 5 μl of the pooled RNA was mixed with 15 μl of the master mix, making a total of 20 μl, and tested as a single sample using the SYBR Green-based RT-qPCR assay. A schematic illustration of the sample pooling strategy is depicted in Fig. 2.

### Assay repeatability and reproducibility

To evaluate the precision of the SYBR Green-based RT-qPCR method, intra- and inter-assay variability was determined using three clinical samples of different Ct values (low, medium, and high). For the intra-assay, the testing was conducted simultaneously in three replicates on the same PCR plate. Under the same conditions and system, three separate PCR runs were performed to assess the inter-assay variation. From both assays, the mean, standard deviation (SD) and coefficient of variation (CV) were calculated independently for each sample. A CV value of less than 10% (intra-assay CV) and 15% (inter-assay CV) was regarded as acceptable.

### Statistical analysis

The cycle thresholds (Ct) for the amplification of each target gene were analysed. The statistical significance of the differences in the different groups of data were analysed by one-way analysis of variance (ANOVA). *P* < 0.05 was considered statistically significant.

### Ethical approval and informed consent

All the methods performed in this study are in accordance with Declaration of Helsinki and have been approved by The Faculty of Medicine, Universitas Indonesia Health Research Ethics Committee with approval number 22-03-0327. The need for informed consent has been waived by The Faculty of Medicine Universitas Indonesia Health Research Ethics Committee (approval number: 22-03-0327) because of residual de-identified nasopharyngeal and oropharyngeal samples which previously identified as positive and negative during routine diagnostic procedures, were used.

## Results

### Primer design and screening for SARS-CoV-2 detection

**Figure 3:**
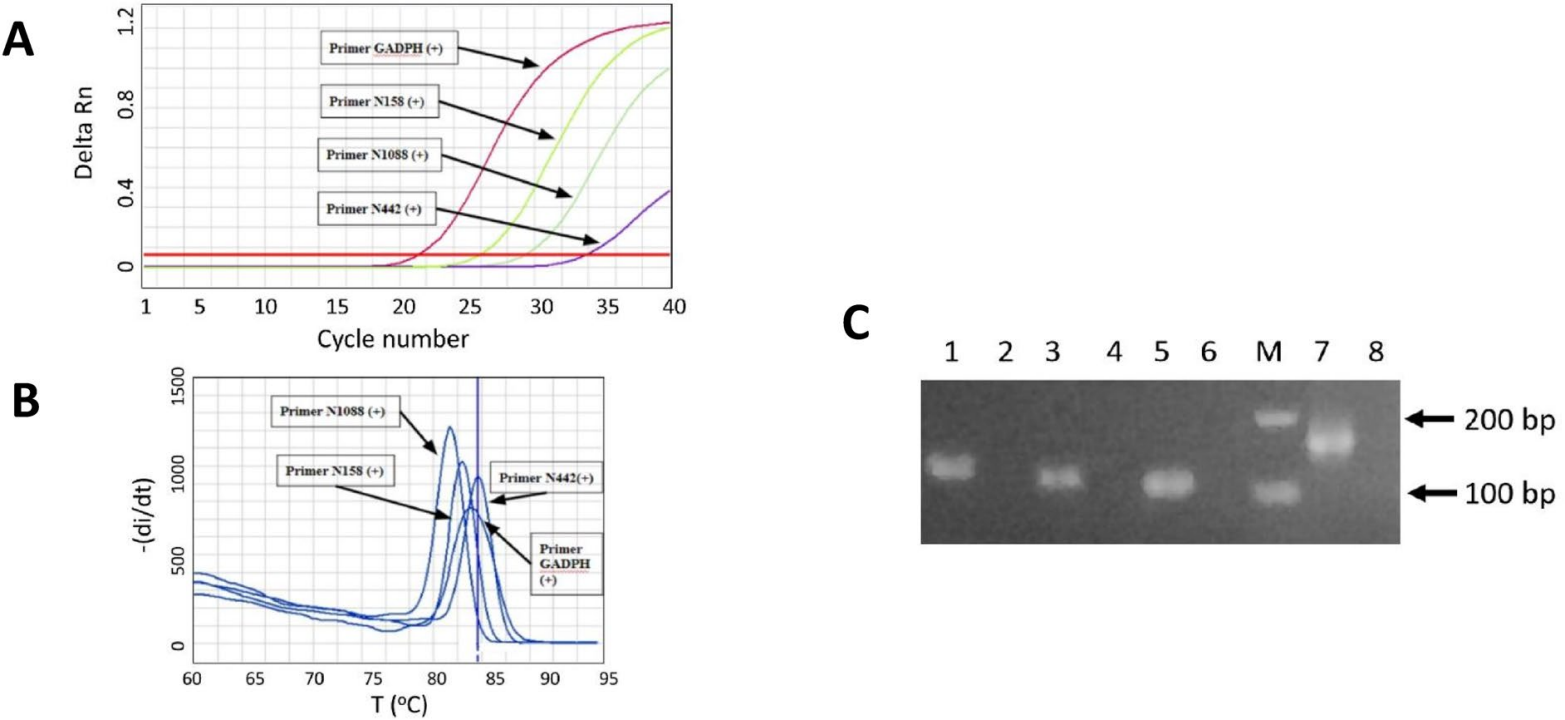
Characteristics of SYBR Green-based RT-qPCR. (**A**) Representative amplification curve of the assays with three different primers targeting N gene and GADPH gene as control primer pair, using a positive SARS-CoV-2 RNA sample; (**B**) melting curve of the products amplified with the N primers and GADPH gene; (**C**) Distinct PCR products amplified with three different primers and GADPH gene (Line 1, 3, 5, and 7) visualized in 2% agarose. Line 2, 4, 6, and 8 indicate negative control of the assays (Nuclease-free water), respectively (original gel is presented in Supplementary Fig. 2C). Note: the arrows mark the molecular weight of 100 bp and 200 bp (Tiangen, China).

We designed primers targeting the N gene of SARS-CoV-2 and selected three pairs for further screening. Additionally, a control primer pair targeting the human housekeeping gene Glyceraldehyde 3-phosphate dehydrogenase (GAPDH), which is commonly employed in our laboratory, was selected for the validation of primer design. All the primer sequences are listed in Table 1, while the location of the N-gene specific primers in the SARS-CoV-2 genome are shown in Fig. 1. In an initial validation experiment to test the primer pair, we employed SARS-CoV-2 RNA from a positive clinical sample. For the SYBR Green-based RT-qPCR protocol, the optimal annealing temperature was set at 60 °C, while optimal primer concentrations were 0.6 µM in a 10 µl of reaction mixture. As a negative control, no-template control (Nuclease-free water) was included for each primer pair. Figure 3 shows the characteristics of the amplification curve for each primer pair or assays after 40 amplification cycles. It is shown that different primer pairs generated different Ct values. No amplification curve was observed for any of the no-template controls (Fig. 3A). This indicates that no unspecific signal occurred during RT-qPCR that might have been caused by primer dimer or contamination. The amplification curve results were then confirmed with melting curve analysis and 2% agarose gel visualization. As shown in Fig. 3B, a clear and distinct melt peak was revealed, corroborating the presence of a single PCR amplicon produced by each assay. Furthermore, single bands of the RT-qPCR amplification segments were also confirmed in the agarose electrophoresis, with different lengths for each primer pair (Fig. 3C). Taken together, these results indicate that by employing the SYBR-Green based RT-qPCR method, all the primers amplified the specific product as expected. Compared to the other two N-gene specific primers (N442 and N1088), N158 generated lower Ct value in the detection of SARS-CoV-2. Essentially, this indicates that N158 assay was more efficient in the amplification of the specific target under the same RT-qPCR conditions. Based on these results, the N158 primer pair was chosen for further study.

**Figure 4:**
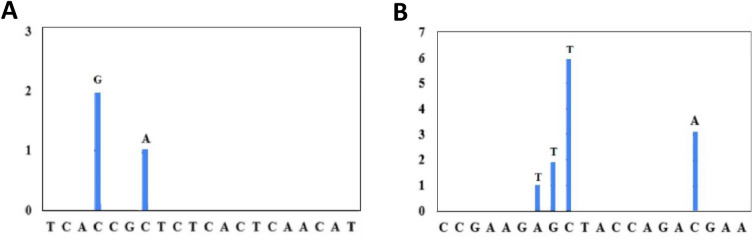
Sequence variants in primer binding regions for N158 primer forward (**A**) and reverse (**B**). A total of 998 SARS-CoV-2 genome sequences from infected individuals of Indonesia, including delta (n = 250), omicron and its subvariants BA.1, BA.2, BA.3, BA.4.1, BA.5, BF.5 and XBB.1 (n = 748) submitted from November 2021 until the end of May 2023 were downloaded from the GISAD database (https://www.gisaid.org/). Using Bioedit Sequence Alignment Editor, each primer was aligned to the genome sequences to calculate nucleotide diversity and investigate mismatches to the genome sequence sets. The position and frequency of each variant/mismatch on the primers was then plotted.

Given the fact that SARS-CoV-2 has continuously evolved, especially during the COVID-19 outbreak, we investigated whether our N158 assay could be compromised by genetic variants or mutations emerging in the SARS-CoV-2 genome. To this end, we analysed genome variants in the primer binding region of the N158 assay using 998 viral sequences reported from Indonesia, including Delta and Omicron variants. Sequence analysis showed that the genome variants in the primer binding region of N158 presented in very low number and existed near the 3’ end (Fig. 4A,B). We found that only two and four variants existed in the forward and reverse primer regions respectively, accounting for 1.5% of the viral sequence analysed. This observation indicates that the primer binding region of N158 are relatively conserved.

### Standard curve and analytical sensitivity of the SYBR Green-based RT-qPCR method

**Figure 5:**
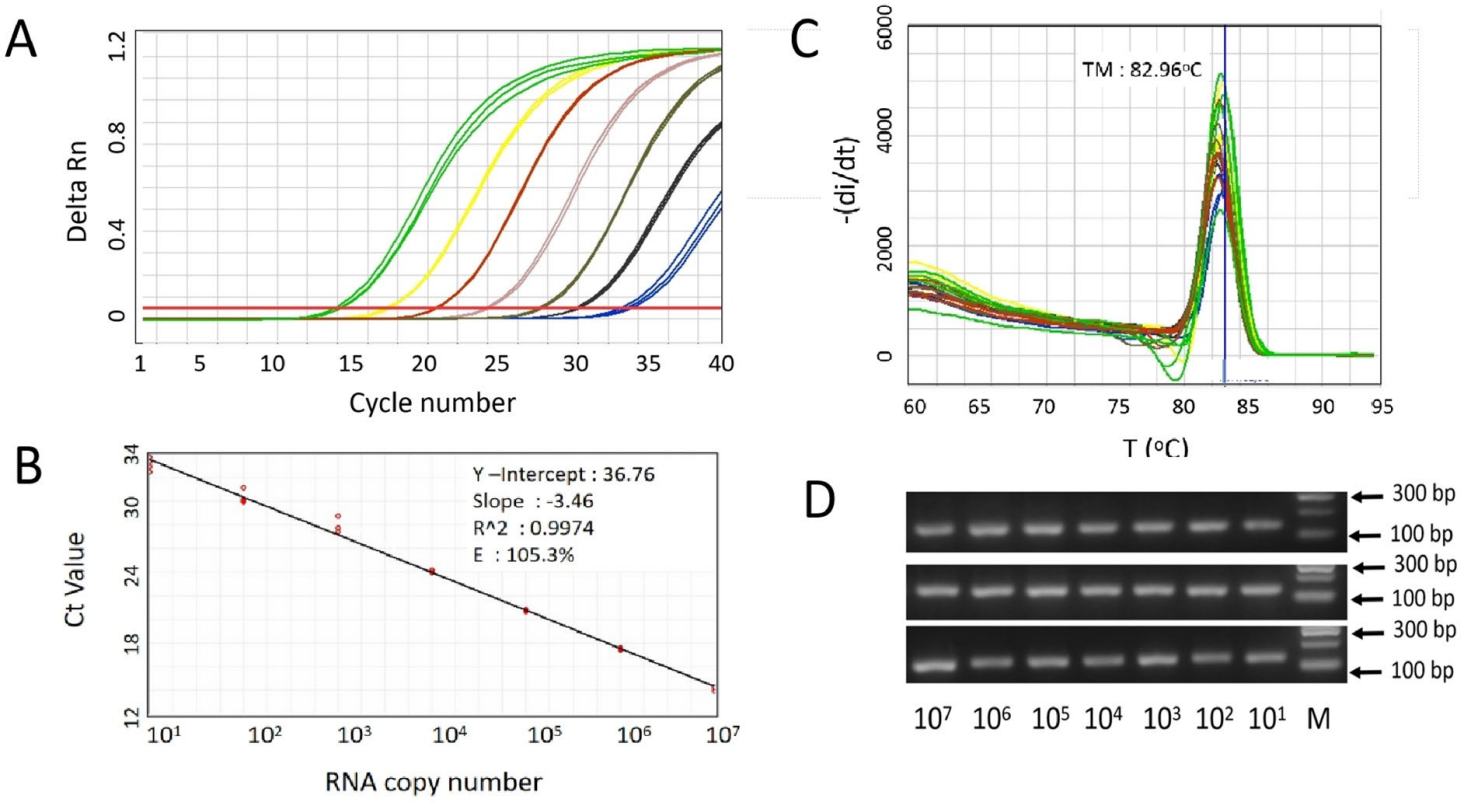
Analytical sensitivity of SYBR Green-based RT-qPCR method for SARS-CoV-2 detection. (**A**) Representative amplification curves for N158 assay targeting N specific gene. Amplification plots (cycle number versus fluorescence) of serially diluted of positive SARS-CoV-2 RNA template (copies/reaction). The RNA concentration of the original sample was measured by the methods described in the previously study (Rahmasari et al., 2022). All seven serially diluted samples, ranging from 10^7^ to 10^1^, were tested in triplicate using the SYBR Green-based RT-qPCR protocol. (**B**) standard curve of N158 assay. RNA copy number is indicated and plotted against the cycle threshold (Ct) value. The coefficient of determination (R2), Y-intercept, slope, and the efficiency (E) of PCR were calculated. (**C**) Corresponding melt curve dissociation for amplified product, unique single peaks with Tm around 82.9 °C were generated in all samples. (**D**) Corresponding RT-qPCR amplicons visualized in 2% agarose gel, amplicon with size of 123 bp were specifically amplified as expected (original gels are presented in Supplementary Fig. 4D).

We then generated standard curves for the SYBR Green-based RT-qPCR protocol by preparing serial ten-fold dilutions of known concentrations of RNA samples (10^7^–10^1^ RNA copy). RT-qPCR was performed under optimal conditions. The results show a positive reaction with all the serial dilutions of the RNA samples (Fig. 5A). RT-qPCR were able to detect the N-gene target down to level of 10 copy RNA. The standard curves show a dynamic linear range across over 7 log units of the RNA copy number. Linear regression analysis revealed that the correlation coefficient (R^2^) was 0.997, with a slope value of -3.46 and amplification efficiency (E) of 105.3% (Fig. 5B). The slope value of -3.46 of the N158 assay indicates that RT-qPCR amplification was efficient^20^. In addition, The single peak with Tm around 82.9 °C were generated in all samples (Fig. 5C) and further verification of the RT-qPCR product with gel electrophoresis shows a correct amplicon with a size of 123 bp (Fig. 5D). Overall, the results obtained from the analytical sensitivity experiment indicate that the performance of our N158 SYBR Green-based assay was sufficiently sensitive for SARS-CoV-2 detection.

### Evaluation of the SYBR Green-based RT-qPCR protocol in clinical samples

**Figure 6:**
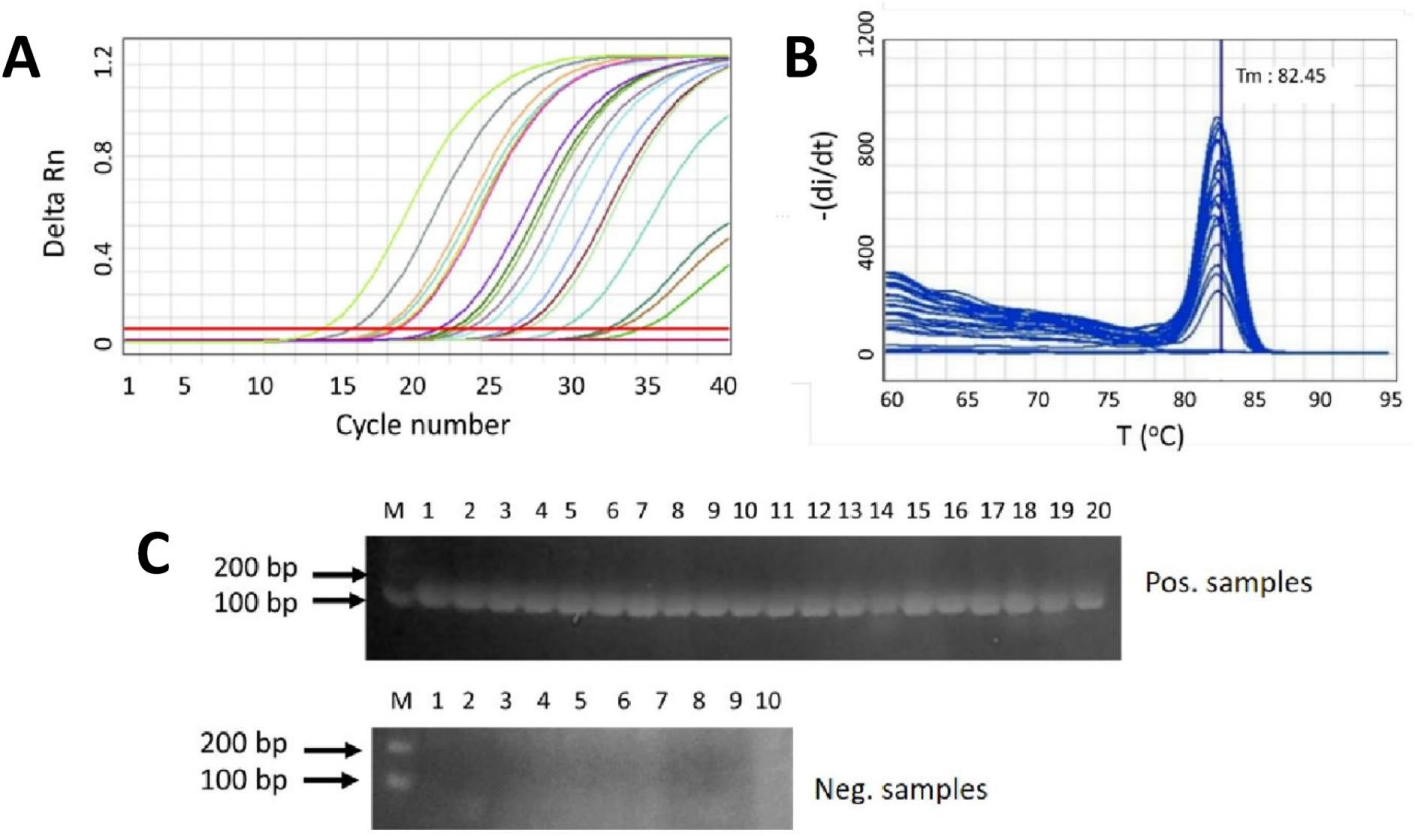
Validation of the SYBR Green-based RT-qPCR method using a total of 124 clinical samples, comprising 82 positive samples and 42 negative samples. (**A**) SYBR Green-based RT-qPCR amplification curves for 20 representative positive samples and 10 representative negative samples. (**B**) Corresponding melt curve dissociation for representative positive and negative samples, unique single peaks with Tm around 82 °C were generated in all positive samples. No nonspecific melt peaks were detected in all negative samples. (**C**) RT-qPCR amplicon with expected size of 123 bp were specifically amplified for positive samples and no unspecifc amplicon were detected in all negative samples (original gels are presented in Supplementary Fig. 5C).

To evaluate our SYBR Green-based RT-qPCR protocol in clinical samples, a set of 124 clinical samples that had been previously tested as positive for SARS-CoV-2 RNA during routine diagnosis were used for the analysis. Ten negative samples were included as negative control. The performance of our SYBR Green-based RT-qPCR protocol was also compared to the commercial TaqMan-based in-vitro diagnostic test kit of Sansure (China). The amplification, melt curve and agarose gel visualization of the clinical samples generated by our new assay are shown in Fig. 6A–C. Table 2 summarizes all the results obtained with RT-qPCR.

**Table 2:** Ct value and melting temperatues Tm of clinical samples tested by TaqMan-based and SYBR Green-based RT- qPCR protocols.

Sample No	Probe based assay (Ct value)		SYBR Green-based assay	
	ORF1ab	N	Ct value	Tm (^o^C)
1	16.30	12.95	13.60	82.45
2	16.82	13.90	15.18	82.24
3	18.00	15.85	18.65	82.24
4	18.62	16.17	18.18	82.24
5	19.05	16.34	17.82	82.24
6	19.36	16.67	18.86	82.24
7	20.08	17.22	18.03	82.24
8	20.74	18.48	18.22	82.45
9	21.30	18.76	21.77	82.24
10	21.98	19.53	22.87	82.03
11	22.20	20.28	22.35	82.03
12	24.71	21.62	24.68	82.17
13	22.98	21.66	26.10	82.24
14	24.71	20.00	22.08	82.33
15	25.72	22.85	24.07	82.03
16	25.72	22.90	26.05	82.24
17	26.92	22.94	26.59	82.45
18	29.96	27.66	29.07	82.24
19	32.41	29.73	31.11	82.24
20	32.53	30.37	33.27	82.24
21	nd	nd	nd	–
22	nd	nd	nd	–
23	nd	nd	nd	–
24	nd	nd	nd	–
25	nd	nd	nd	–
26	nd	nd	nd	–
27	nd	nd	nd	–
28	nd	nd	nd	–
29	nd	nd	nd	–
30	nd	nd	nd	–
31	32.20	31.72	34.88	81.95
32	31.28	28.86	29.08	81.95
33	27.79	27.45	32.34	82.16
34	30.04	28.08	32.05	82.38
35	31.00	29.83	33.59	82.59
36	30.04	31.57	34.07	82.16
37	nd	nd	nd	–
38	32.54	30.87	35.54	82.16
39	nd	nd	nd	–
40	nd	nd	nd	–
41	nd	nd	nd	–
42	32.39	32.82	34.71	81.95
43	30.47	32.49	34.47	81.09
44	nd	nd	nd	–
45	nd	nd	nd	–
46	nd	nd	nd	–
47	20.16	20.43	23.44	82.38
48	nd	nd	nd	–
49	27.75	26.79	29.77	81.30
50	32.31	31.06	34.56	82.38
51	31.51	31.32	35.86	82.16
52	33.46	29.73	30.21	82.16
53	nd	nd	nd	–
54	30.07	29.69	31.21	82.16
55	30.54	30.12	34.16	81.95
56	nd	nd	nd	–
57	nd	nd	nd	–
58	28.09	29.46	33.54	81.95
59	20.63	20,89	22.37	82.16
60	30.33	29.33	29.93	82.16
61	20.60	21.15	23.84	82.16
62	31.35	30.07	33.61	81.73
63	33.26	32.22	34.29	81.52
64	31.16	29.29	34.13	81.73
65	27.08	27.19	30.47	81.73
66	24.43	25.32	29.04	81.73
67	28.58	27.41	30.14	81.73
68	31.52	30.98	35.82	81.95
69	33.80	33.47	33.38	81.95
70	32.31	31.16	34.42	81.73
71	nd	nd	nd	–
72	nd	nd	nd	–
73	17.6	18.06	23.13	82.31
74	nd	nd	nd	–
75	20.29	21.36	25.88	82.52
76	nd	nd	nd	–
77	25.5	27.6	31.00	82.09
78	17.8	19.8	21.91	82.52
79	29.70	31.20	32.81	82.09
80	19.20	20.05	23.30	82.09
81	27.70	30.11	31.81	82.09
82	30.12	29.55	32.04	82.09
83	nd	nd	nd	–
84	nd	nd	nd	–
85	nd	nd	nd	–
86	nd	nd	nd	–
87	nd	31.22	34.22	82.52
88	19.08	20.08	25.40	82.09
89	17.88	19.30	23.17	82.30
90	15.90	17.00	21.50	82.09
91	nd	nd	nd	–
92	27.11	28.40	31.28	82.74
93	nd	nd	nd	–
94	nd	nd	nd	–
95	19.77	21.80	22.69	82.52
96	nd	nd	nd	–
97	–	31.59	35.86	82.16
98	nd	nd	37.69	78.72*
99	nd	nd	nd	–
100	nd	nd	nd	–
101	–	34.46	35.31	81.95
102	nd	nd	nd	–
103	nd	nd	nd	–
104	nd	nd	37.81	77.21*
105	31.08	28.50	30.22	81.95
106	nd	nd	nd	–
107	nd	nd	nd	–
108	27.75	26.62	32.53	82.16
109	nd	nd	nd	–
110	27.47	27.36	33.62	82.38
111	19.06	19.07	24.36	82.38
112	16.06	17.47	22.56	82.38
113	20.13	20.62	26.84	82.38
114	15.12	16.02	21.80	82.38
115	27.17	27.55	33.04	81.95
116	18.26	18.69	23.40	82.16
117	23.88	24.76	28.21	82.16
118	27.48	29.74	33.76	81.73
119	16.69	17.31	22.16	82.38
120	28.82	30.47	33.14	82.16
121	27.22	29.54	33.25	82.16
122	29.99	31.86	32.12	82.16
123	30.99	29.34	32.40	82.16
124	21.20	21.25	23.74	82.59

nd: not detected.

*: unspesific.

Overall, the results showed that the SYBR Green-based RT-qPCR could detect 100% of the positive samples. Moreover, it was able to recognize 100% of the negative samples correctly. These results are in 100% concordance with the commercial TaqMan-based RT-qPCR assay. In addition, the Ct value obtained from our new assay was comparable with that obtained from the TaqMan assay for each individual positive sample. When confirmed with melt curve analysis, the positive samples generated clear unique peaks with Tm around 82 °C, indicating the presence of single and specific RT-qPCR amplicon (Fig. 6B). In the negative samples, no peaks were generated. Further confirmation of the sensitivity and specificity of the SYBR Green- based RT-qPCR assay were also made using agarose gel electrophoresis. Once more, only single bands were obtained from the positive samples with the expected size, and no bands were observed in the negative samples (Fig. 6C). These results demonstrated that our SYBR Green-based RT-qPCR assay was sensitive and specific.

We further evaluated the accuracy and reproducibility (intra- and inter assay) of the SYBR Green-based assay. To this end, three clinical samples of different Ct values (low, medium, and high) were tested repeatedly. For the intra-assay, the Ct values were obtained from three tests of the samples in triplicate. However, for the inter-assay, this was determined based on the Ct values obtained from the triplicate samples in three consecutive tests. The overall inter- and intra-assay coefficients of variation (CV) calculated were 2.35% (ranging from 1.81 to 2.99%) and 3.45% (ranging from 1.73 to 6.02%) respectively (Table 3). Such variable experimental values are considered acceptable, demonstrating that our SYBR Green-based RT-qPR is reproducible.

**Table 3:** Intra- and inter-reproducibility of SYBT Green-based N158 assay.

Sample	Intra-assay variation^a^		Inter-assay variation^b^	
	Cycle threshold (Ct)^C^	CV (%)	Cycle threshold (Ct)^c^	CV (%)
Low Ct	15.9 ± 0.29	1.81	16.20 ± 0.98	6.02
Medium Ct	19.78 ± 0.45	2.26	20.24 ± 0.53	2.59
High Ct	32.29 ± 0.97	2.99	3.45 ± 2.2	1.73
Overall CV (%)^c^	2.35 ± 0.60		32.41 ± 0.56	

^a^Intra-assay variation was determined on three replication of clinical samples of different Ct values and analysed in the same PCR run.

^b^Inter-assay variation was calculated on values obtained in three separate PCR run.

^c^Mean ± standard deviation (SD) CV : coefficient of variation.

### RT-qPCR results of pooling the RNA of the samples

We finally performed RT-qPCR testing on the pooled RNA samples using our newly developed SYBR Green-based protocol. A total of 70 pooled RNA samples (each pool contain one positive and four negative samples) were prepared for the analysis.

**Figure 7:**
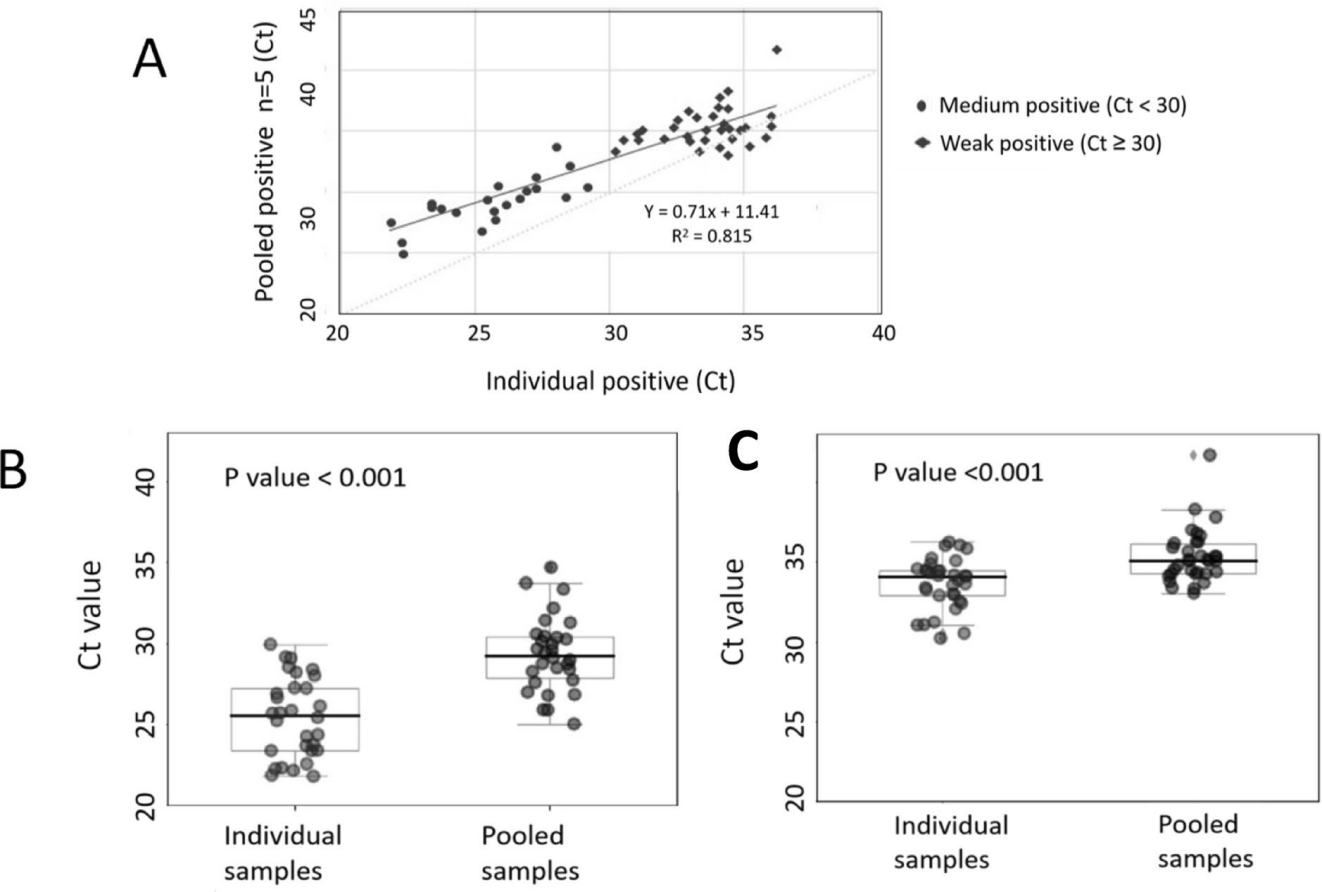
Comparison of Ct value of pooling strategy. Seventy previously identified positive RNA samples were used, including 36 samples that had tested as weak positives (Ct ≥ 30) and 34 samples that had tested as medium positives (Ct < 30). (**A**) Ct values distribution of pooled (1/5 samples) and individual samples of medium and weak positive measured with the SYBR Green-based RT-qPCR protocol. (**B**) Mean Ct value (median, range) of medium positive and (**C**) weak positive. Original data is presented in Supplementary Fig. 7, respectively.

The positive sample set included 34 medium positive (Ct value < 30) and 36 weak positive (Ct value ≥ 30) samples, based on their individual testing results. In the analysis of 5-sample pooling within the medium positive group, all 34 pool samples tested exhibited 100% concordance with the individual positive samples. However, within the weak positive group, 7 positive samples went undetected after pooling, all with Ct > 33. These discrepancies accounted for 23% of the samples with Ct > 33 (7 out of 33). The distribution of RT-qPCR Ct values for the 63 positively-detected pooled samples and individual samples is depicted in Fig. 7A. Regression curve analysis demonstrated a strong positive linear correlation between the Ct values of the individual and pooled samples with R2 value of 0.815. For further analysis, we also compared the Ct values (median, range) of pooled and individual samples within the weak positive and medium positive sample sets separately (Fig. 7B,C). The mean Ct values of the pooled and individual samples for the medium positive samples were 29.34 ± 2.31 and 25.42 ± 2.47, respectively. For the weak positive samples, the mean Ct values of the pooled and individual samples were 35.34 ± 1.71 and 33.63 ± 1.60, respectively. As expected, when comparing the pooled and individual Ct values, there was an increase of 3.93 ± 1.63 cycle threshold (Ct) for the medium positive sample and 1.71 ± 1.83 Ct for the weak positive sample, indicating a decrease in viral loads. These differences were found to be statistically significant (*p* < 0.001).

## Discussion

During the COVID-19 pandemic, demand for molecular diagnosis testing grew quickly worldwide. At present, different RT-qPCR kits used to detect SARS-CoV-2 in patient samples are being widely developed. Since TaqMan-based RT-qPCR was recommended by WHO as the gold standard for SARS-CoV-2 detection, most RT-qPCR assays commercially available on the market are based on TaqMan probe. This method is extremely effective, but not accessible to all laboratories, especially in developing countries. In this study, we have developed a low cost SYBR Green-based real time RT-qPCR assay as an alternative molecular protocol for the detection of SARS-CoV-2 in clinical samples. For this purpose, we designed a new primer pair that targets the nucleocapsid protein (N) gene of SARS-CoV-2.

Our N158 primer were designed based on the sequence from an early 2021 SARS-CoV-2 isolate (MZ026854.1) from Indonesian patients. Since viruses constantly evolve, especially during an outbreak, mutation can emerge, including within the target regions of the primer. When this occurs within the primer region, the mutation could compromise the sensitivity and specificity of RT-qPCR assay^21^. To investigate this issue, we conducted genetic analysis to establish whether and how frequently mutation had already occurred within the N158 primer binding region of SARS-CoV-2. Through *in-silico* analysis using a set of 998 SARS-CoV-2 genome sequences from infected individuals in Indonesia, including Delta and Omicron variants, our observations show that there is only a very low prevalence of genome variation in the N158 primer binding regions. This indicates that these regions are highly conserved. Our observation is line with the genetic analysis of coronavirus showing that the N gene of SARS-Cov-2 is conserved and stable. When compared to the SARS-CoV-2 S gene, for example, which has 76% amino acid similarity to the SARS-CoV S gene, the SARS-CoV-2 N gene has 90% amino acid similarity to the SARS-CoV N gene. This indicates that only a few mutations had occurred over time in the coronavirus N gene^23–25^. In general, high conservation of the coronavirus genome is facilitated by the proofreading mechanism of the non-structural protein 14 (nsp14) of the viruses. Nsp14 provides activity to remove nucleotides misincorporated by the RNA-dependent RNA polymerase (RdRp) during viral RNA synthesis and thus generates faithful replication of the large (~ 30 kb) coronavirus genome^22,25,26^.

Indeed, mutation or mismatch that occurs in primer binding regions does not necessarily affect assay sensitivity. This is particularly true when the genetic variants are present on the left end or 5 prime half of a primer. Specifically, in relation to the sensitivity of diagnostic primers, the 5’ half of a primer is not as important as its 3’ half. In our study, the variants in the forward and reverse primer regions were located near the 5’ end. Therefore, this might not pose a major concern in relation to the sensitivity of our new N158 assay. With regards to analytical sensitivity, our SYBR Green-based assay has been shown to be sensitive for the detection of SARS-CoV-2. With the dynamic range of the assay spanning 7 log units, it was able to detect N molecules up to 10 copy number. The N158 assay has a sensitivity that is similar than that reported in studies targeting the same SARS-CoV-2 N gene^27,28^.

Using a set of clinical samples, we validated our newly developed SYBR Green-based RT-qPCR assay. All the samples found to be positive by the commercial TaqMan method were confirmed by the SYBR-Green method. All the negative samples tested by TaqMan assay were also found to be negative in the SYBR-Green assay. These results were confirmed by agarose electrophoresis and melting curve analysis. From these two approaches, no primer-dimer or non-specific products were observed in either the positive or negative samples. This indicates that regarding clinical performance, our SYBR Green-based assay was specific and demonstrated similar performance to the TaqMan method in detecting SARS-CoV-2 directly from clinical samples. In addition, the reported intra- and inter-assay variations regarding the Ct values were very small (< 3% for intra-assay and < 4% for inter-assay), suggesting that the N158 assay could generate reproducible results. Therefore overall, besides being sensitive, our SYBR-Green-based RT-qPCR was shown to be specific and reproducible. We believe that our SYBR-Green assay could be used as replacement for the existing TaqMan RT-PCR for effective detection of SARS-CoV-2 in clinical practice.

Apart from its low cost and no need for probe design and synthesis, one of main advantages of SYBR Green-based assay compared to TaqMan-based assay is that it is a simpler approach to designing primers and optimization procedures^29^.In other words, working with the SYBR Green method is cheaper and easier than using TaqMan. Theoretically, probe-based methods such as TaqMan are superior to SYBR Green-based assay. The power of the TaqMan method is due to its unique design based on oligonucleotide double-labelled probes that significantly increase the sensitivity and specificity of the assay. In contrast, SYBR Green is an unspecific dye. It can intercalate into any double-stranded DNA (dsDNA) such as primer dimers and emits a false positive signal. This unspecific signal can finally lead to a reduction in the performance of SYBR Green-based assay^30,31^. However, false positive results due to SYBR properties can be avoided by incorporating melting curve analysis at the end of each PCR run. By performing this step, the accuracy of the SYBR Green-based methodology can be assured. In this study, as mentioned above, our SYBR Green-based assay was comparable to TaqMan in the detection of SARS-CoV-2 in the clinical samples. It has been demonstrated that with optimisation of the SYBR Green method, including primer design, its performance and quality could be as good as the TaqMan method.

Commercial COVID-19 RT-qPCR test kits use a variety of SARS-CoV-2 RNA gene targets. Most of the tests target the envelope (E), nucleocapsid (N), spike (S), RNA-dependent RNA polymerase (RdRp) and *ORF1* genes of SARS-CoV-2. In addition, they commonly target multiple genes of the virus through the multiplexing strategy^32^. Apart from its capability to simultaneously detect more than one target in a single analysis, multiplex assay allows accurate diagnosis even if critical mutations occur in the primer binding site of one of the gene targets. This aspect is relevant, because the SARS-CoV-2 genome is constantly mutating, and mutation can occur throughout the virus genome. In this study, we developed an SYBR Green-based assay which targets the N gene of SARS-CoV-2. We utilized the N gene since it is highly conserved and the most sensitive target for SARS-CoV-2^28,33^. The superiority of the N gene with respect to sensitivity is due to its presence in higher amounts in samples compared to other targets. It has been reported that the N gene exists in more abundance as messenger RNAs in coronavirus and subgenomic of mRNA of the N gene are generated during virus replication^34^. Here we used a single gene target of the N gene. US CDC protocol utilizes two primer sets to two different regions of N gene targets, which are tested in two separate tubes. Both targets, namely N1 and N2, have shown similar performance in the detection of the virus, with no differences between Ct or measurable viral genome copies^35,36^. Our previous study^19^ also demonstrated that singleplex amplification of the N gene was equally sensitive and specific in comparison to the commercial TaqMan assay. This suggests that only a single amplification of N sequences could be used for diagnosis of COVID-19, thus reducing testing costs. Furthermore, although our N158 primer were designed based on the sequences from the initial isolate of an Indonesian patient, our N primer are still suitable with respect to assay sensitivity for the detection of SARS-CoV-2. It proved high genetic stability of the N gene in the coronavirus family.

We also evaluated the pooling strategy for efficient detection of SARS-CoV-2. When the disease prevalence is lower, such as in the COVID-19 post-pandemic era, pooling tests might be an alternative approach to effectively enhance detection capability and reduce the testing burden. Previously, pooling strategy has been successfully used for the detection of other infectious pathogens, including the hepatitis B virus, hepatitis C virus and human immunodeficiency virus type-1 among blood donors^37,38^. For COVID-19, a pooling strategy could reduce costs by 69% and it requires tenfold fewer tests^39,40^.

Three different approaches to pooling tests can be taken: (i) the collection of more than one nasopharyngeal swab in a universal container or VTM; (ii) the mixing of several VTM samples from different patients prior to RNA extraction (pooling of VTM); and (iii) the mixing of several extracted RNA into one sample before RT-qPCR (pooling of extracted RNA). In this study, we chose the third option, as described in previous studies^41,42^.

Our results demonstrate, in the medium positive group (Ct < 30), there is a 100% concordance between pooling and individual testing. It indicates that 5-sample pooled testing is feasible for samples with a high or medium viral load. However, in the weak positive group, the concordance decreases. Approximately 23% of borderlines samples with Ct value > 33 failed to be detected as positive, indicating that pooling may increase the risk of false negative results when dealing with very low viral loads. Nonetheless. these results underscores the potential advantage of the pooling approach for mass screening testing or epidemiological surveillance purposes.

Furthermore, most pooling studies for SARS-CoV-2 detection have primarily relied on the TaqMan assay. Our study demonstrates that SYBR Green-based RT-PCR can be a viable alternative for implementing the pooling strategy in the detection of SARS-CoV-2. As mentioned earlier, the SYBR Green-based real-time PCR assay is more cost-effective than the TaqMan-based assay. The use of SYBR Green has reduced the material costs for diagnosis by half when compared to probe-based methods^43–45^. In the context of Indonesia, for in-house assays, the SYBR Green-based method typically costs between US $1.0–1.5 per reaction, while the cost of a TaqMan probe can range from US $2–3. These estimates cover the cost of all reagents, excluding consumables used in a PCR reaction. However, it's important to note that these costs can vary significantly based on factors such as the specific supplier, brand, and the scale of the experiment. Additionally, when compared to conventional PCR, the SYBR method is also more cost-effective, with a fourfold reduction in expenses^43^. According to our calculations, the overall cost per sample for conventional PCR, excluding consumables, ranges from US $3–4. This relatively higher cost for conventional PCR is due to the need for post-PCR analysis which involves agarose electrophoresis. This post-PCR step requires additional reagents such as agarose, TBE Buffer, Gel-Red (DNA dye), Gel Loading Dye, and DNA Ladder.

Based on our experiences, conventional PCR, when using high-quality Taq polymerase enzyme, can yield results of comparable sensitivity to those obtained with SYBR Green-based methods. Despite the higher initial investment of real-time PCR instruments, real-time PCR offers numerous advantages when compared to conventional PCR for long-term application. This is especially evident when handling high-throughput samples, such as during the SARS-CoV-2 pandemic. Real-time PCR is also less time-consuming, as it eliminates the need for additional post-PCR steps, and it is less labor-intensive. Although SYBR Green-based RT-qPCR methods reduce the cost of reagents per test, the high cost of the equipment remains a barrier to entry, especially in low-income countries, posing limitations to its widespread adoption.

The sample size is one limitation of our study, primarily due to budget constraints. A more extensive testing of clinical specimens is required to further evaluate the diagnostic reliability of our new N158 assay both in individual samples and pooled samples. In addition, validation of our SYBR Green-based RT-qPCR assays using other human pathogenic coronaviruses could be the basis for future studies.

## Conclusion

In summary, our newly developed SYBR Green-based real time RT-qPCR assay has been proven to be sensitive and specific and could serve as a simple approach for the detection of SARS-CoV-2 in clinical samples. Furthermore, the approach of combining up to 5 samples for SARS-CoV-2 detection using SYBR Green-based RT-qPCR analysis offers a high diagnostic yield. However, it's important to consider that when dealing with samples having a very low viral load (Ct > 33 in our study), pooling may increase the risk of missing positive cases. Nonetheless, the sample pooling strategy could offer a cost-effective approach for mass screening tests and epidemiological surveillance purposes, especially in the COVID-19 post-pandemic era.

### Supplementary Information

Supplementary Information 1.

Supplementary Information 2.

## Data Availability

All data generated or analysed during this study are included in this published article [and its supplementary information files].
