# Supplementary material for: Development and validation of cost-effective SYBR Green-based RT-qPCR and its evaluation in a sample pooling strategy for detecting SARS-CoV-2 infection in the Indonesian setting
Source: Sci Rep. 2024 Jan 20;14:1817. doi: 10.1038/s41598-024-52250-w (PMC10799953; doi:10.1038/s41598-024-52250-w)
Supplement: Supplementary file 1 — Supplementary Information 1. [file 41598_2024_52250_MOESM1_ESM.pdf]

Supplementary Figure 2C

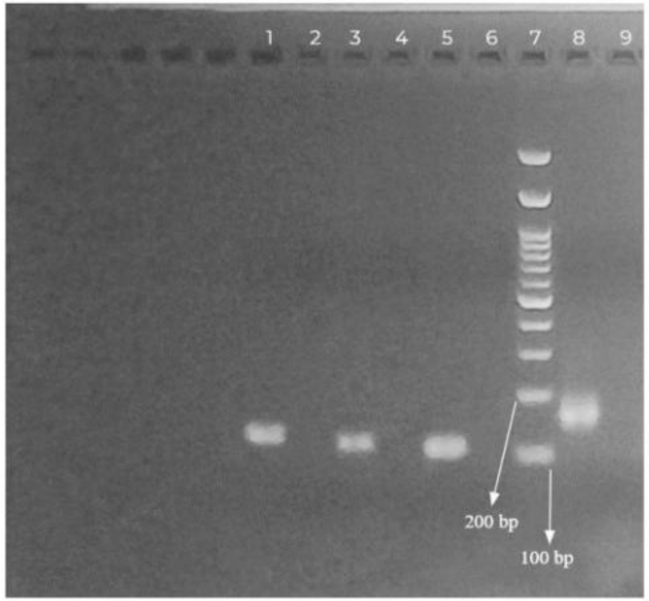

labeled-raw figure
(original)

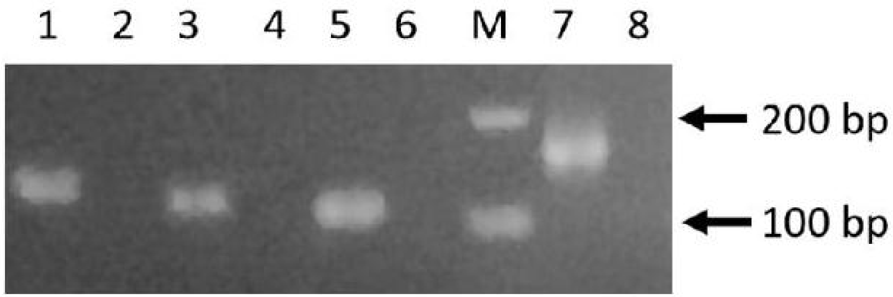

edited figure
(Journal)

Supplementary Figure 4D

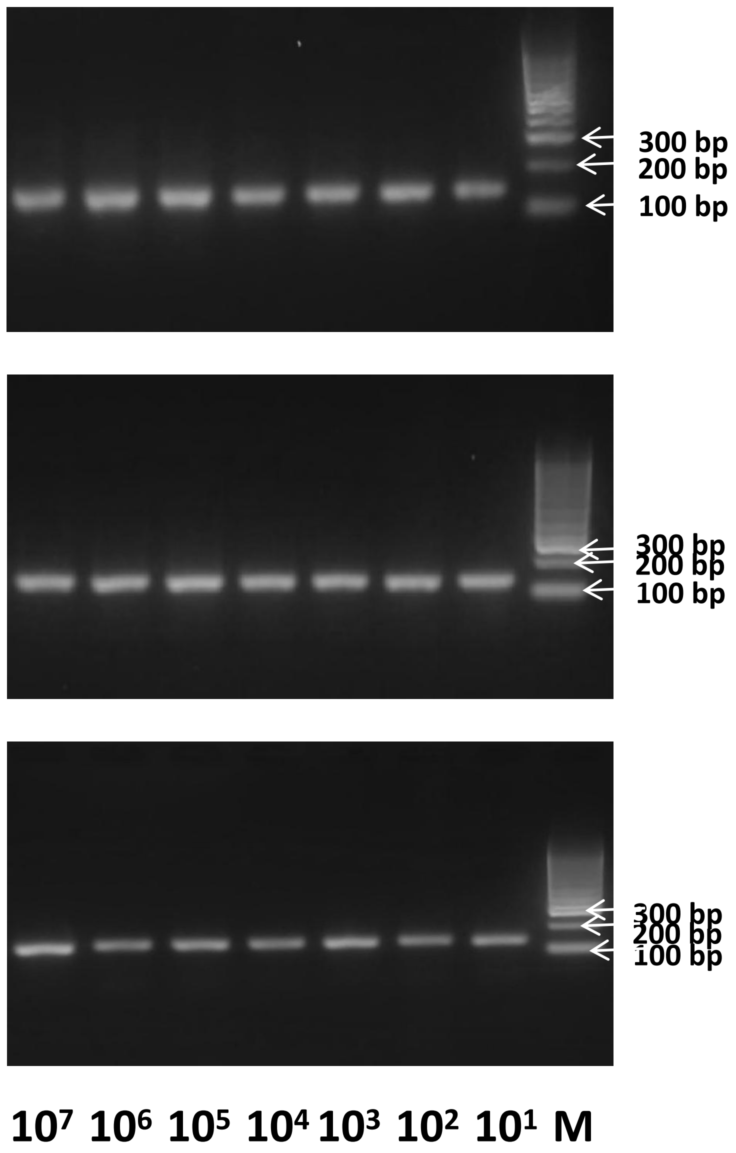

labeled-raw figure
(original)

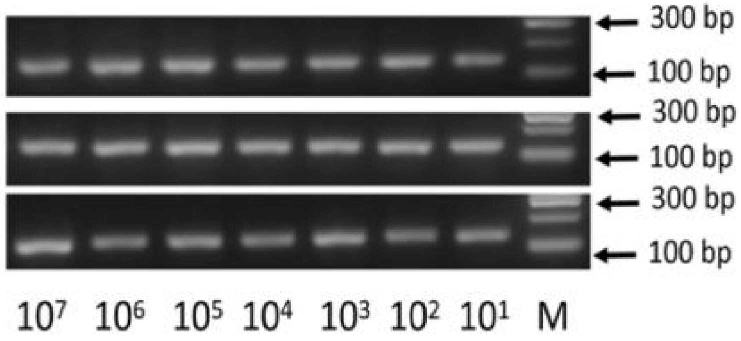

edited figure
(Journal)

Supplementary Figure 5C

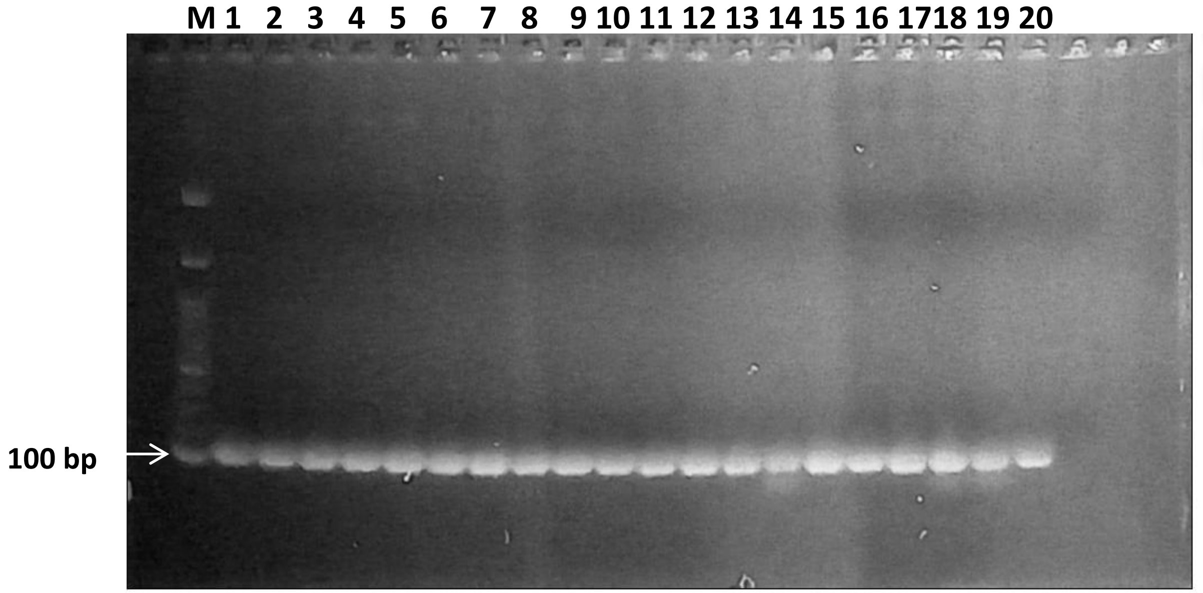

labeled-raw figure
(positive sample, original)

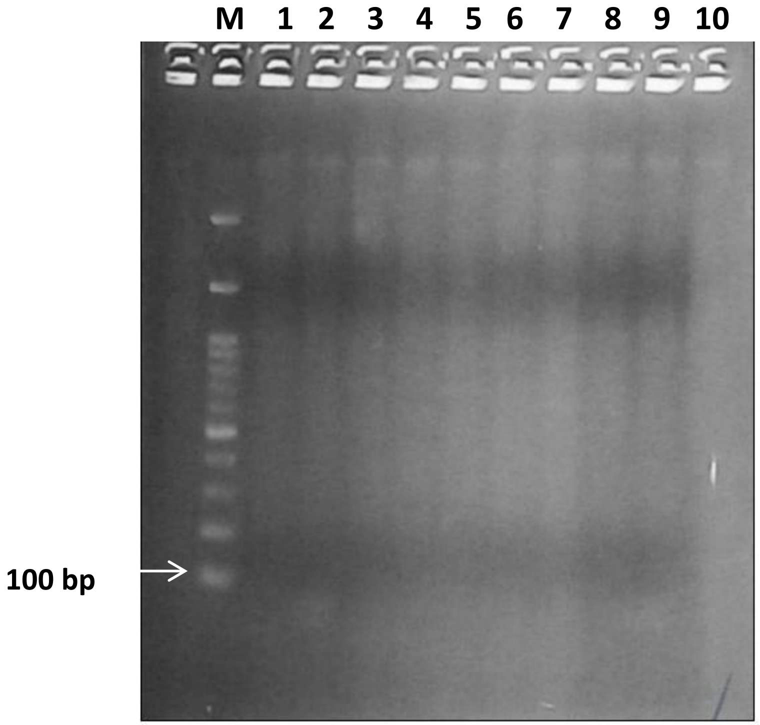

labeled-raw figure
(negative sample, original)

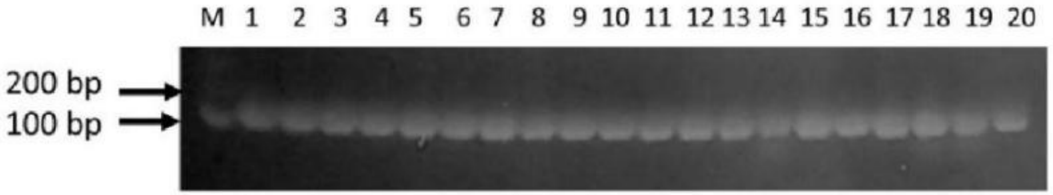

edited figure
(positive sample, Journal)

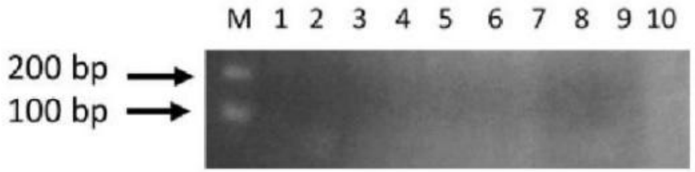

edited figure
(positive sample, Journal)

Supplementary Figure 7

Tabel S1. 5-Sample pooling

Sample no.	Category	Ct value		
		Individual	5-Sample pool	Delta Ct
1	Medium	25.22	26.83	1.61
2	Medium	21.86	27.58	5.72
3	Medium	23.38	29.1	5.72
4	Medium	30.52	34.25	3.73
5	Medium	31.06	34.25	3.19
6	Medium	34.42	36.8	2.38
7	Medium	25.85	30.57	4.72
8	Medium	27.25	30.35	3.1
9	Medium	24.26	28.39	4.13
10	Medium	22.32	25.02	2.7
11	Medium	26.12	28.97	2.85
12	Medium	25.68	28.48	2.8
13	Medium	27.23	31.27	4.04
14	Medium	25.71	27.73	2.02
15	Medium	28.52	32.16	3.64
16	Medium	23.38	28.75	5.37
17	Weak	31.04	34.78	3.74
18	Weak	32.89	34.54	1.65
19	Medium	26.89	30.12	3.23
20	Medium	23.69	28.73	5.04
21	Medium	22.26	25.9	3.64
22	Medium	26.64	29.56	2.92
23	Medium	25.42	29.41	3.99
24	Medium	28.01	33.71	5.7
25	Weak	36.04	36.23	0.19
26	Weak	36.23	41.66	5.43
27	Weak	35.06	35.34	0.28
28	Weak	33.84	36.24	2.4
29	Weak	33.00	34.11	1.11
30	Weak	34.12	37.79	3.67
31	Weak	34.43	38.28	3.85
32	Medium	29.15	30.43	1.28
33	Medium	28.38	29.65	1.27
34	Weak	32.93	36.64	3.71
35	Weak	34.88	35.13	0.25

Tabel S1. continue

36	Medium	29.08	31.41	2.33
37	Weak	32.05	34.33	2.28
38	Weak	35.21	33.75	-1.46
39	Weak	34.07	36.99	2.92
40	Medium	23.74	26.78	3.04
41	Weak	35.54	nd	-
42	Weak	34.71	nd	-
43	Weak	34.47	35.14	0.67
44	Weak	34.56	34.37	-0.19
45	Weak	35.86	nd	-
46	Weak	30.21	33.36	3.15
47	Weak	31.21	35.05	3.84
48	Weak	34.16	35.04	0.88
49	Weak	33.54	34.23	0.69
50	Medium	29.93	34.68	4.75
51	Weak	33.61	35.05	1.44
52	Weak	34.29	35.64	1.35
53	Weak	34.13	33.66	-0.47
54	Weak	35.82	34.46	-1.36
55	Weak	33.38	33.31	-0.07
56	Weak	34.42	33.04	-1.38
57	Medium	24.36	26.98	2.62
58	Weak	32.53	35.89	3.36
59	Weak	36.01	35.36	-0.65
60	Medium	22.56	29.94	7.38
61	Medium	21.8	28.26	6,46
62	Weak	33.04	nd	-
63	Medium	23.4	30.25	6.85
64	Medium	28.21	33.33	5.12
65	Weak	33.76	nd	-
66	Medium	22.16	25.89	3.73
67	Weak	36.14	nd	-
68	Weak	33.25	36.15	2.9
69	Weak	36.12	nd	-
70	Weak	32.4	35.31	2.91

nd : not detected
